# Response surface optimized removal of cefixime from wastewater samples using magnetic ferric oxide nanoparticles

**DOI:** 10.1186/s13065-025-01635-7

**Published:** 2025-10-06

**Authors:** Rana W. Gaber, Amr M. Mahmoud, Sarah S. Saleh

**Affiliations:** 1 Analytical Chemistry Department, Faculty of Pharmacy, October University for Modern Sciences and Arts (MSA), 6th October City, 11787 Egypt; 2 Pharmaceutical Analytical Chemistry Department, Faculty of Pharmacy, Cairo University, Kasr-El Aini Street, Cairo, 11562 Egypt

**Keywords:** Adsorption, I-optimal, ECO scale, AGREE, GAPI

## Abstract

Cefixime is a broad-spectrum cephalosporin antibiotic that is orally administrated to treat bacterial infections diagnosed from mild to moderate ones. Since cefixime is considered a widely used antibiotic in many countries, it was important to develop a method for its removal from water as a part of water purification using an adsorption technique with different nanoparticles. In order to optimize its removal conditions, a fractional factorial design was applied to screen experimental factors including pH, contact time, and amounts of nanoparticles. A three-factor, two-level I-optimal design was designed using Derringer’s desirability algorithm, in which the optimal removal conditions of cefixime were found to be Fe_3_O_2_ dose (13 mg/L), pH (5.9), and contact time (180 min) at room temperature. An HPLC method was developed for monitoring the adsorption process using a Kinetex C_18_ stationary phase (100 mm L × 4.6 mm i.d., particle size 5 μm, USA), and a mobile phase consisting of phosphate buffer (adjusted to pH 6.8) and methanol in the ratio of 75: 25 which was pumped at a flow rate of 1 mL/min at room temperature with UV detection at 288 nm. The proposed HPLC method was validated according to ICH guidelines and was assessed using the greenness tools Analytical Eco-scale (AES), GAPI, and AGREE. The removal and analysis procedures were successfully applied to simulated wastewater samples containing cefixime with a bias of 1.9%. The method can be easily applied for large-scale water treatment.

**Supplementary Information:**

The online version contains supplementary material available at 10.1186/s13065-025-01635-7.

## Introduction

Cephalosporins are beta-lactam semi-synthetic antibiotics widely used in veterinary and human bacterial infection therapy. The World Health Organization (WHO) stated that cephalosporins are considered one of the most significant and high-priority antimicrobials for human medicine [1]. The core structure for cephalosporins was first obtained from the fungus *Cephalosporium acremonium* [2]. It works by binding proteins to the microbial membrane leading to cell wall synthesis inhibition which causes cell death and lysis [3]. Cephalosporins are considered less allergenic and less vulnerable to inactivation by beta-lactamase enzyme when compared to other antibiotics such as penicillins. Cephalosporins interrupt microbial growth by acting against gram-negative and gram-positive bacteria [4, 5].

Cefixime is authorized by the U.S. Food and Drug Administration (FDA) as it is considered a broad-spectrum oral administrated cephalosporin antibiotic to cure mild to moderate bacterial infections. Cefixime is considered very efficient against a wide range of bacteria such as *Escherichia coli*,* Staphylococcus pneumonia*,* Hemophilus influenza*,* Neisseria gonorrhea*, and *Streptococcus pyogenes* [6, 7]. Moreover, it is efficient against bacteria causing sore throat, middle ear infections, urinary tract infections, tonsillitis infections bronchitis, gonorrhea, laryngitis, and pneumonia. Cefixime is found to be a primary candidate for bacterial treatment therapy as it has a wide safety profile and good efficacy [8]. Moreover, it is an inexpensive, safe, and effective oral choice for the cure of pharyngitis and enteric fever in children. Its chemical formula is shown in Fig S1. Cefixime has been determined by some techniques such as high-performance liquid chromatography (HPLC) [9–12], high-performance thin-layer chromatography (HPTLC) [13] spectrophotometry [14–16], fluorimetry [17] and voltammetry [18, 19].

The antibiotic residuals present in the aquatic environment show a major global threat as they exert a negative impact on organisms. Furthermore, it has been reported that antibiotics can easily migrate to the drinking water which can lead to serious health problems due to drug resistance that can be evolved [20, 21]. It was found that the source of water and soil contamination with antibiotics are wastes from the drug manufacturing industry, hospitals, domestic activities, and livestock farming due to animal wastes [22]. Therefore, the assessment of the water quality is important and represents a great challenge as antibiotics were found to be in trace amounts [23]. Since cephalosporins are widely used antibiotics, they can make their way to different types of water [24, 25]. There are a lot of different methods that have been developed for water treatment such as ion exchange [26], electrolysis [27], reverse osmosis [28], air stripping, photocatalytic degradation [29–31], ozonation process [32] and adsorption [33]. Despite the presence of several techniques for water treatment, it was found that the technique of adsorption was one of the most effective routes for antibiotics removal, especially cephalosporins, as it was found to be more applicable, eco-friendly, fast, inexpensive, and useful in removing the soluble and insoluble contaminants [34]. On the other hand, other methods were found to be more costly, and time-consuming, while other treatment routes can lead to the production of secondary products that may contaminate the water [35].

Adsorption can be performed via different mechanisms including the exchange of ions in which ions exchange happens between the contaminants and the adsorbent material. Chemisorption is when a bond is formed between the contaminant and the adsorption material, while physical adsorption is where the contaminant is attached to the surface of the adsorbents by Van Der Waals weak force. Several adsorbents can be used such as ferric oxide [36], magnesium oxide [37], graphene oxide [38], and titanium dioxide [39] nanoparticles.

The nano-sized magnetic nanoparticles have super-paramagnetic properties, a high saturation field, and a high irreversible field. Ferric oxides have a strong influence on magnetic fields due to their very small crystal surface and size [40]. Ferric oxide nanoparticles have appealing characteristics in the field of environment remediation and pollutant adsorption due to their high ability of adsorption, easy preparation, high stability, convenient operation, and facile recovery and recycling [41]. They were able to remove many compounds such as arsenic and methyl red pollutants [42]. They showed a fast adsorptive rate and large adsorption capacity for these contaminants [41]. Graphene oxides have been stated to show a high ability of adsorption in the first class due to high surface area, thermal and mechanical properties, excellent electrical conductivity, and good chemical stability [43]. However, it was found that graphene oxide applications in environmental remediation are limited due to its aggregation in an irreversible way in solution by π–π and van der Waals interactions which make it poor reliable, non-specific, and low repeatable [44]. Titanium dioxide nanoparticles are considered semiconductors that have unusual structures, electronic, magnetic, and chemical properties. It has been proved that the titanium oxide nanoparticles have excellent photocatalytic properties [45]. Therefore, it has a wide range of applications such as photo-catalysis, synthesis of inorganic membranes, solar cells, air and water purification [46]. Therefore, these nanoparticles were studied for their efficiency in cefixime removal.

Many factors can affect the technique of adsorption for example nanoparticle concentration, contact time, pH, and drug concentration [47], which should be tuned to achieve the optimum remediation conditions. The optimization of adsorption factors can be studied as one factor at a time [48], or using the design of experiment [49].

Response surface methodology (RSM) is a design of experiment (DOE) procedure that includes several statistical and mathematical methods for developing empirical models that use the behavior of data sets provided to estimate the outcomes, known as prediction. RSM aims to establish a relationship between a response and the levels of several input variables or factors that impact it through the proper design and analysis of trials [50]. I-optimal design is an RSM design that reduces the prediction variance integral over the entire design space. Hence, it was selected to study and optimize the factors of the treatment process [51].

This study aims to develop an optimized method for the removal of the antibiotic Cefixime from wastewater using various nanoparticles. The removal of cefixime was performed via an adsorption technique with the aid of different nanoparticles such as reduced graphene oxide, titanium dioxide, and ferric oxide as effective adsorbents. To ensure removal efficiency, a low-cost, eco-friendly technique, a chromatographic method for determining Cefixime in wastewater was developed and validated. Cefixime removal was optimized using an I-optimal design to get the optimum conditions of treatment while minimizing the number of experimental runs, time, and cost.

## Experimental

### Instrument

The HPLC system used was Waters Alliance 2690 (Waters, UK) equipped with a Waters 996 PDA detector and pump with a low-pressure mixing system and a vacuum degasser was used for chromatographic pressure. Kinetex C18 (4.6 mm i.d. × 100 mm L, particle size 5 μm, USA) was used to carry out the separation. The system was supplied with a binary solvent delivery pump, photodiode array detector, and autosampler. pH meter (Jenway, 3505, UK) was used to adjust the pH of the mobile phase. A Sonicator (Batron SA Soni-clean-120T, Australia) and a Shimadzu electronic balance (model number: ATY224), (Japan) were employed. Centrifugation was done by refrigerated centrifuge (Sigma 2–16 K Bench-top). The design of experiment and data analysis were done by using the Design-Expert program 10.0. 64-bit.

### Materials and reagents

The Cefixime (CEF) standard was generously provided by Hikma Pharma (6th of October city, Egypt) with a purity of 99.5%. Ferric oxide, titanium dioxide, and reduced graphene oxide were provided from Nano Gate (Mokaatam, Cairo, Egypt). KH_2_PO_4_, HCl, Na_2_HPO_4,_ and NaOH were purchased from El-Nasr Company (Cairo, Egypt). HPLC grade methanol (Sigma-Aldrich, Germany).

### Standard solutions

12.5 mg of CEF was precisely weighed and moved to a 25 mL volumetric flask, then methanol was used to dissolve the CEF to obtain a stock solution with a concentration of (0.5 mg/mL). Two mL of stock solution was transferred into a 50 mL volumetric flask to prepare the working solution and completed with distilled water to obtain a working solution of concentration (20 µg/mL). The volume was adjusted to the mark using the mobile phase. Different aliquots of the working solution corresponding to 100–1000 µg of CEF were precisely weighed and transferred into a series of 10 mL volumetric flasks, and then the solvent was used to adjust the volume to the mark to prepare working solutions in the concentration range of 10–100 µg/mL. The intended chromatographic conditions were applied. The relationship between the corresponding drug concentration and mean peak area (A) was constructed and represented as a calibration curve as shown in Fig. S2.

### Synthesis of the nanoparticles

Nano Gate reported that the following steps were used for the synthesis of the Nanoparticles (NPs): (1) Black powder of Ferric oxide (particle size < 10 nm) was prepared by the chemical co-precipitation principle to generate plain magnetic particles. 9.94 gm of ferrous chloride tetrahydrate powder (FeCl_2_·4H_2_O) and 23.64 gm of ferric chloride hexahydrate powder (FeCl_3_·6H_2_O) were dissolved into 300 mL of distilled water. The temperature was raised to 80 °C, and 80 mL of ammonium aquation (25%) was added under high-speed stirring (350 rpm) to reach pH 10.0, this reaction took place for 45 min after that the magnetic nanoparticles were separated by centrifugation at 5000 rpm for 15 min and washed by a mixture of ethanol + deionized water several times. (2) Titanium dioxide (particle size < 10 nm) was synthesized by precipitation from a homogenous solution using titanium (VI) isopropoxide as a precursor. (3) Reduced graphene oxide was provided as black sheets in powder form, its length ranging from 1 to 3 μm and thickness from 1 to 5 nm. It was prepared by chemical reduction of graphene oxide using sodium borohydride. Further details of the preparation of both titanium dioxide and reduced graphene oxide are mentioned in Table S1.

### Characterization of ferric oxide nanoparticles

The microstructural properties of ferric oxide nanoparticles were investigated using a high-resolution transmission electron microscope (HR-TEM) and X-ray diffraction (XRD). HR-TEM analyses were performed by JEOL JEM-2100 microscope operating at an accelerating voltage of 200 kV. A Gatan digital camera, Erlangshen ES500, was used to facilitate capturing images, enabling a detailed visualization of the nanoparticles’ crystalline structure and morphology. X-ray diffraction (XRD) measurements were performed using the XPERT-PRO Powder Diffractometer system to define the nanoparticles’ crystalline phases and structural parameters. The diffraction parameters were conducted over a 2θ range of 20° to 80°, utilizing a fine step size of 0.001° and Cu kα radiation with a wavelength of 1.54614°.

### Chromatographic conditions

The HPLC elution was performed on Kinetex C_18_ (4.6 mm i.d. × 100 mm L, particle size 5 μm, USA). The mobile phase consists of phosphate buffer (adjusted to pH 6.8) and methanol in the ratio of 75:25 v/v. Phosphate buffer was prepared by dissolving 3.01 gm of KH_2_PO_4_ and 5.04 gm of Na_2_HPO_4_ in 1000 mL of distilled water. The flow rate used for pumping the mobile phase was 1 mL/min at room temperature. The sample was injected at a volume of 20 µL. The quantification was computed by using the peak area with UV detection at 288 nm.

### Screening experiment

A fractional factorial design was applied to screen experimental factors including different types of nanoparticles, nanoparticles amount, pH, and contact time. In a set of 50-mL beakers, 20 mLs of CEF working solution was transferred to a beaker, then different doses of ferric oxide nanoparticles (0.005 and 0.015 gm) at different pH values (5.0 and 7.0). The beakers were left for different contact times (60 and 180 min). The experiments were performed in the absence and presence of TiO_2_ and reduced graphene oxide of 10 mg each. After the contact time, the external magnet was used to separate the loaded solution which was then subjected to centrifugation at 10,000 rpm for 15 min. Then, the supernatant was isolated to be used for HPLC measurements.

### Optimization experiment

According to the preliminary results, a three-factor, two-level I-optimal design was utilized to select the optimum conditions of CEF adsorption. Nineteen runs were designated including 4 center points and carried out experimentally. In a set of 50-mL beakers, 20 mLs of CEF working solution (20 µg/mL) were transferred to a beaker, and conditions were settled according to the design in which different doses of ferric oxide nanoparticle (0.005 and 0.015 gm) at different pH (5.0 and 7.0). The beakers were left for different contact times (60 and 180 min). All the samples were centrifuged at 10,000 rpm for 15 min and the supernatant was then isolated for HPLC measurements to estimate the percentage of CEF removal.

The lack-of-fit p-value, the probability values (*P* < 0.005), and the coefficient of determination R were applied to evaluate the fitted quadratic model quality. With the application of Derringer’s desirability function (D), the optimum condition was observed and determined at which the optimum response was detected. The regression model equation was then used to predict the design space of responses.

### Adsorption kinetics and isotherm studies

Kinetic studies were carried out using 0.013 g/L of ferric oxide nanoparticles at pH 5.9 with a CEF initial concentration of 20 µg/mL. The left concentration of CEF was measured at different time intervals (15–180 min) till the residual CEF amounts for two successive measurements almost overlapped. Pseudo-first and pseudo-second order kinetics models were used to fit the adsorption.

#### Kinetics data

Langmuir and Freundlich adsorption isotherm models were used to demonstrate CEF’s adsorption by magnetic ferric oxide nanoparticles. The effect of initial CEF concentration on the adsorption capacity of the ferric oxide nanoparticles was studied by changing CEF concentrations within the range (5.0–25.0 µg/mL) at a fixed amount of ferric oxide nanoparticles (0.013 g/L) and pH 5.9 for 180 min.

## Results and discussion

This work aims to develop an optimized adsorption method of CEF for its removal from wastewater using ferric oxide nanoparticles.

### Characterization results of ferric oxide nanoparticles

**Fig. 1:**
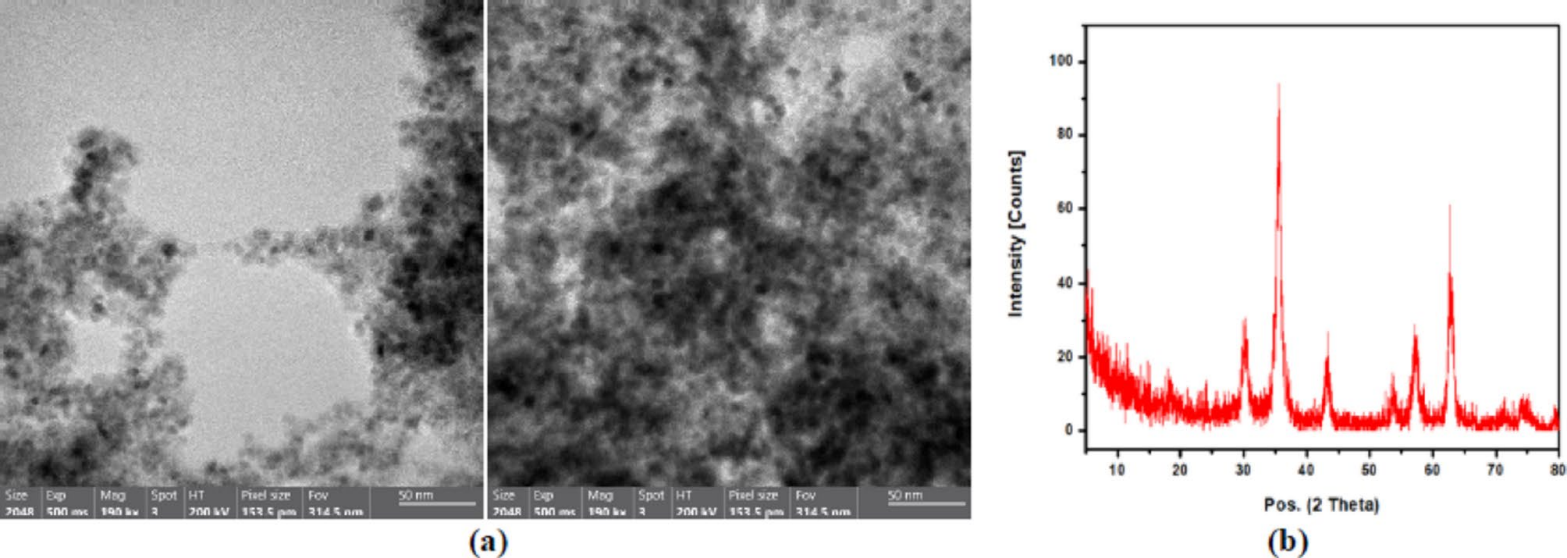
**a** TEM image and **b** XRD pattren of Ferric oxide nanoparticles

The ferric oxide nanoparticles’ morphological features were analyzed by using HR-TEM as shown in Fig. 1a at a magnification 190,000x. The captured images showed the creation of nano-scale particles with an average diameter less than 10 nm that confirm the synthesis of fine magnetite nanoparticles. In addition, aggregated roughly spherical nanoparticles were formed with high surface contact due to its dipole-dipole interactions that is favorable for adsorption purpose by providing large surface area to volume ratio. The nanoscale dimensions provide higher active sites suitable for adsorbent binding and facilitate rapid mass diffusion to the particle surface, which is an important feature in environmental remediation and water treatment applications.

XRD technique was used to investigate the crystalline structure of ferric oxide nanoparticles and the corresponding diffraction pattern, as shown in Fig. 1b. The major diffraction peaks were observed at 2θ positions near 30.2°, 35°, 43.3°, 53°, 57° and 62.9°, which shows good agreement with the standard magnetite phase. No secondary or impurity peaks were detected, confirming the purity of the nanoparticles. The intensity and sharpness of the peaks indicate a well-crystallized material, and the broadening of peaks shows the presence of nanosized nanoparticles. The nanoscale size and high degree of crystallinity improve the surface characteristics of magnetite, allowing the nanoparticles to be eligible for adsorption applications.

### Development and optimization of chromatographic method

**Fig. 2:**
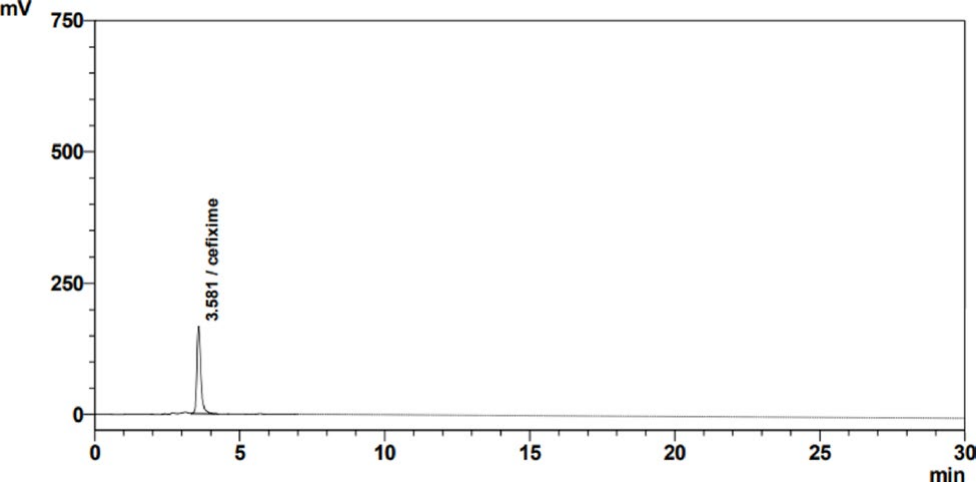
Chromatogram of CEF using C_18_ as a column and a mobile phase consists of methanol and phosphate buffer (pH 6.8) in the ratio of 25:75 v/v

To get the optimal requirements for the proposed HPLC method, the effect of different variables was necessary to be tested. Different types of stationary phases were tried (Zorbax C_8_ and Kinetex C_18_ columns), where the C_18_ column showed the best peak symmetry. Furthermore, different solvents were tried as mobile phases (methanol and acetonitrile), and it was found that the acetonitrile caused noisy peaks, but the methanol-eluted peaks were sharp. Furthermore, when methanol was mixed with phosphate buffer at a ratio of (25:75, v/v), better resolution and sharper peaks were obtained. Different pH values for the buffer were tested but they led to unsuccessful adsorption and broad peaks, but pH 6.8 was found to be optimum. Different flow rates in the range of 0.5mL/min to 1.5 mL/min were examined, however, the best compromise for retention time and flow rate value was 1 mL/min which gave a symmetric peak. The detection was done at 288 nm in which the injected volume was at 20 µL and the retention time was 3.6 ± 0.1 min, as shown in Fig. 2.

### Validation of the HPLC method

The developed method was subjected to validation testing according to ICH guidelines regarding the following parameters: linearity, limit of quantification (LOQ), limit of detection (LOD), robustness, precision, accuracy, specificity, and system suitability. The validation parameters were tested on prepared solutions using the standard cefixime and listed in Table 1.

**Table 1:** Validation parameters of the proposed HPLC method

Parameters	HPLC	Reference values^*^
Concentration range (µg/mL)	10–50	
Correlation coefficient (r)	0.9994	
Slope	58,346	
Intercept	130,568	
SD of residuals from line	39,852	
LOD (µg/mL)	2.254	
LOQ (µg/mL)	6.830	
Accuracy (Recovery % ± SD)	101.71 ± 2.02	
Precision (RSD)		
Intraday	0.771	
Interday	1.322	
t_R_, min	3.916 ± 0.05	
Tailing factor (T)	0.97	T ≤ 2, T = 1 for symmetric peak
Capacity factor (K’)	1.77	K’= 1–10 acceptable
Plates number (N)	3214	*N* > 2000
Height equivalent to theoretical plate (HETP; cm plate ^−1^)	0.04	The smaller the value, the higher the column efficiency

### Linearity

Standard solutions were prepared and injected in the range of 2–50 µg/mL, and the calibration curve was attained by plotting concentration versus peak area. The linear regression analysis was used to evaluate linearity with the aid of the least square method. A good linear relationship over the concentration range (10–50 µg/mL) was shown by the calibration curve’s linear regression data with a correlation coefficient greater than 0.999.

### LOQ and LOD

The limit of quantitation (LOQ) and limit of detection (LOD) were valued at a signal-to-noise ratio of 10:1 and 3:1, respectively. LOQ was equal to 6.83 µg/mL, while LOD was found to be equal to 2.25 µg/mL.

### Robustness

Robustness was examined by deliberately altering the experimental conditions. Three levels of different experimental conditions such as percentage of methanol [25], flow rate (1.00 mL/min), and detection wavelength (288 nm) were examined. The impact of changing one factor at a time regarding the experimental conditions on the assay results was examined where RSD < 2. The developed method is considered robust as there was no peak area or retention time change observed.

### Precision

The intra-day precision (repeatability) for the method was examined by analyzing three standard solutions of cefixime at concentrations of 10, 20, and 30 µg/mL on the same day. The same three standard solutions were subjected to inter-day testing on three successive days to determine the intermediate precision. The data obtained for precision studies (intra-day and inter-day) were expressed as relative standard deviation (RSD%), and it was confirmed that the method was precise.

### Accuracy

The analysis of six blind concentrations of cefixime was done, and the recovery was found to be in the range of 98–102%, while the mean recovery was found to be 101.71 and the standard deviation (± SD) was less than 2 indicating the accuracy of the method.

### System suitability

The parameters evaluation like tailing factor, theoretical plates, resolution and retention time examined the system suitability and its outcomes showed that the developed method is proper for the intended purpose.

### Design of experiment (DOE) for the adsorption process

#### Screening (fractional factorial design)

**Fig. 3:**
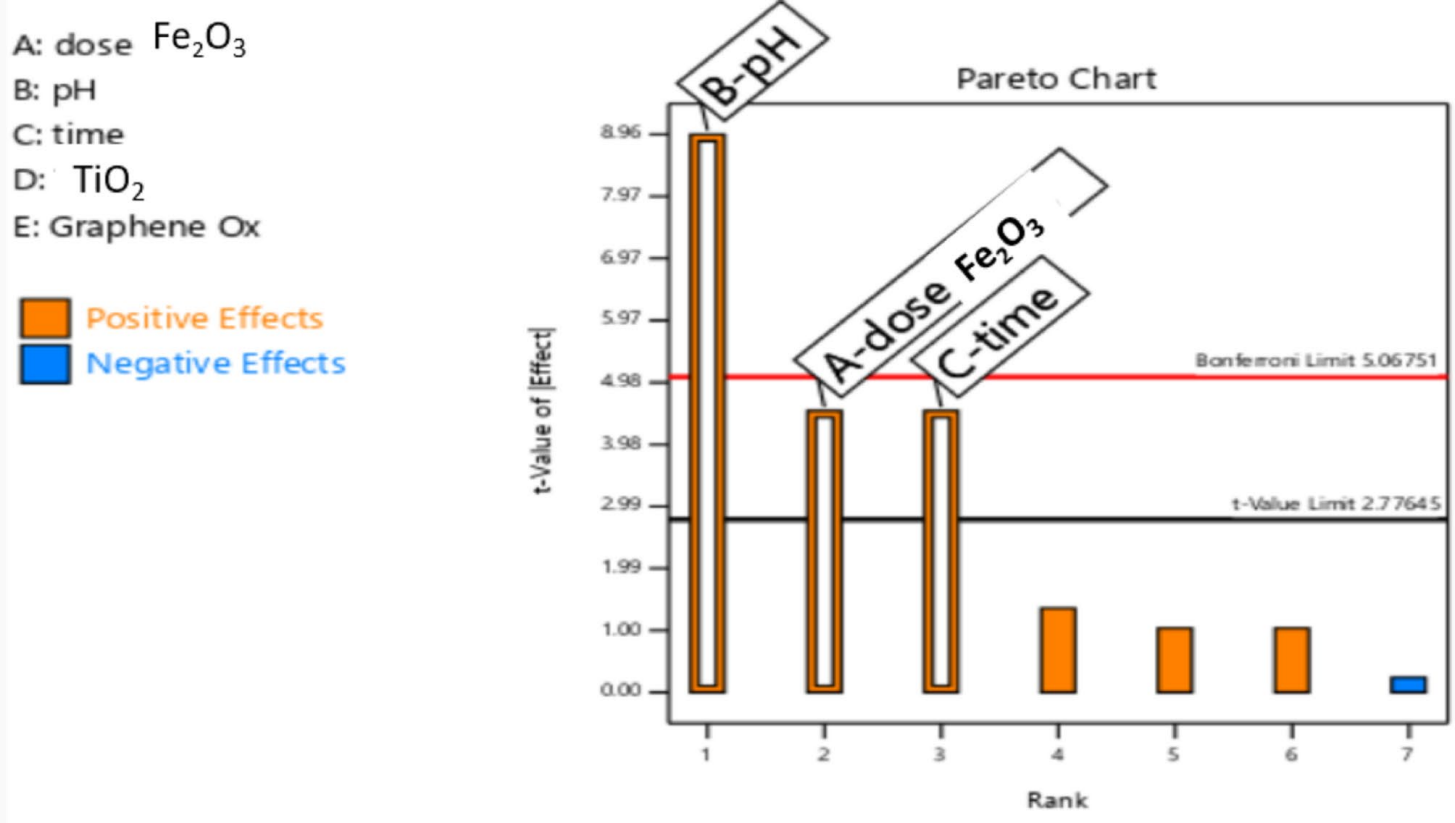
Pareto chart of the fractional factorial design showing the significant factors: **a** dose of ferrous oxide, **b** pH, and **c** contact time

Since there were several factors required to be examined whether they affect the adsorption process of cefixime or not, the effectiveness of the factors of interest and their impact on the response were examined by performing a screening factorial design. The examined factors were different nanoparticles such as ferric oxide, titanium dioxide, and reduced graphene oxide, pH, and contact time. Variance analysis was done to find out the effect of each variable, and it was found that the p-value for the dose of ferric oxide nanoparticles, pH, and contact time were less than 0.05 which means that those were the significant factors; while the p-value for the addition of titanium dioxide and reduced graphene oxide were higher than 0.1 which means that they were insignificant as shown in the Pareto chart in Fig. 3.

According to the fit statistics, the R-squared (R^2^) was found to be 0.9680. The predicted R^2^ (0.8722) is in reasonable agreement with the adjusted R^2^ (0.9441) as the difference is less than 0.2. Adequate precision was calculated to be equal to 18. The collected data was analyzed by the regression model analysis which helps in production between the independent variables and responses represented by the second-order polynomial equation:

1 $$ {\text{R}}\% = 46.63 + 7.13\;{\text{A}} + 14.13\;{\text{B}} + 7.13\;{\text{C}} $$

where the R% is the cefixime removal percentage, A is the dose of ferric oxide, B is the pH and C is the contact time in minutes.

Thus, a study on the optimum response for CEF removal/adsorption was done by employing an I-optimal design and enhancing the CEF removal percentage by choosing the most affecting variables, which were found to be the dose of ferric oxide nanoparticle, pH, and contact time. The diagnostic graphs including the residual plots are shown in Fig S3.

### Optimization (I-optimal design)

Response surface methodology (RSM) was chosen as the statistical procedure to study the factors that can affect CEF adsorption. The I-optimal design was used to set up an experimental design to help in studying the effective adsorption parameters that give an optimum adsorption response by using minimal experimental runs and minimizing the cost, time, and effort as well as determining these parameters’ importance. The I-optimal design takes into consideration the quadratic and linear effects and the interaction effects between the selected variables on the intended response. The selected variables under investigation were the dose of ferric oxide, pH, and contact time. These significant factors are determined and examined with two levels low and high (− 1 & +1) for each factor. The three factors with their two levels and the experimental runs number are presented in Table 2. The 19 runs were performed randomly to reduce any biased responses with four center points and 5 vertexes. The collected data was analyzed by the regression model analysis that helps in the production of a relation between the responses and factors which are illustrated by the following second-order polynomial equation as follows:

2 $$ \begin{aligned} {\text{R}} & = 76.66 + 4.36\;{\text{A}} + 7.66\;{\text{B}} + 8.99\;{\text{C}} - 1.67\;{\text{AB}} - 0.6171\;{\text{AC}} \\ & \quad + 5.36\;{\text{BC}} - 2.42\;{\text{A}}^{2} - 37.71\;{\text{B}}^{2} + 1.65\;{\text{C}}^{2} \\ \end{aligned} $$

where the removal percentage of CEF is expressed by R, A is the dose of Fe_2_O_3_ nanoparticles in g/L, B is the pH and C is the contact time in minutes. The sign before the numbers reflects the variable effect in which the direct effect for A, B, C, BC, and C^2^ is indicated by a positive sign while the inverse effect for AB, AC, A^2^, and B^2^ is indicated by a negative sign.

**Table 2:** The experiments and results of I-optimal design used for optimization of the removal percentage of CEF

Run	Dose of Fe_2_O_3_	pH	Contact time (min)	*R*%
1	0.015	5.5	180	88.3038
2	0.01	3.5	180	35.2
3	0.005	3.5	120	23.11
*4*	0.01	7.5	180	52.322
5	0.015	7.5	120	47.46
6	0.01	3.5	60	30.4787
7	0.015	3.5	120	35.81
8	0.01	7.5	60	34.2376
9	0.01	5.5	120	76.89
10	0.01	5.5	120	76.3139
11	0.015	5.5	60	72.77
12	0.005	7.5	120	39.7141
13	0.01	5.5	120	75.9
14	0.005	5.5	180	82.33
15	0.005	5.5	60	60.1165
16	0.015	7.5	60	33.88
17	0.005	7.5	60	30.22
18	0.01	5.5	120	77.19
19	0.015	3.5	60	32.44

According to the fit statistics, the regression model values were found to be within acceptable limits. The (R^2^) was found to be 0.9975 which shows that the quadratic model explained 99.75 of the total variances. The predicted R^2^ (0.9736) is in reasonable agreement with the adjusted R^2^ (0.9943) where the difference is less than 0.2 which estimates the predictive power of the model and shows that the model is a good fit with a polynomial equation. Adequate precision was calculated to be equal to 49.23.

According to the model ANOVA in Table 3, the F value was found to be 311.59 and the p-value was found to be < 0.0001, which reveals that the model is significant with a non-significant lack-of-fit. The ***p*** and ***F*** values were computed for each variable and its interaction. The diagnostic graphs, including the residual plots, are shown in Fig. S4.

**Table 3:** ANOVA statistics for quadratic model of optimization design

Source	Sum of squares	df	Mean square	F-value	*p*-value	
Block	461.86	1	461.86			
*Model*	8168.95	9	907.66	311.59	< 0.0001	Significant
A-NP conc	177.00	1	177.00	60.76	0.0001	
B-pH	320.43	1	320.43	110.00	< 0.0001	
C-Contact time	451.17	1	451.17	154.88	< 0.0001	
AB	16.26	1	16.26	5.58	0.0502	
AC	2.13	1	2.13	0.7297	0.4212	
BC	82.26	1	82.26	28.24	0.0011	
A^2^	20.53	1	20.53	7.05	0.0327	
B^2^	4824.63	1	4824.63	1656.23	< 0.0001	
C^2^	9.21	1	9.21	3.16	0.1187	
*Residual*	20.39	7	2.91			
Lack of Fit	19.90	5	3.98	16.10	0.0595	Not significant
Pure Error	0.4944	2	0.2472			
*Cor total*	8651.20	17				

### Interaction of variables and model validation

According to the regression analysis of the models, the correlation between the predictor variables and the response was determined by the polynomial equations. The change in response variable is estimated by the coefficient value for each factor while keeping the other factors constant. For the quadratic terms, it is observed that the large coefficient term for pH means that it is the dominant variable and has the highest effect among the other factors, followed by the dose of ferric oxide particles which in turn has more impact than the contact time. It is proved that the isoelectric point of ferric oxide is very important as it affects the adsorption of surfactants on nanoparticles surface. The changes in pH affect the ionization of the cefixime drug, the pH was tested in the range of 3.0–8.0. At pH 3.0 it was shown low % of cefixime adsorption which is caused by the positive charge on the surface of ferric oxide which makes it unable to bind to cefixime (positively charged). By increasing the pH, it was found that the adsorbed amount of the tested drug increased exceptionally, that’s because the ferric oxide became negatively charged which allowed its binding with the positively charged cefixime by polar interaction such as hydrogen binding with the C2 hydrogen of the imidazolium ring and ion-dipole interaction.

The effect of pH is positive which means that the response is directly affected by the pH as the response increases by the increase of pH until it reaches a certain point then the response decreases. Furthermore, the positive coefficient of the other factors, which are the dose of nanoparticles and contact time indicates they both have a synergetic effect in the same way. In other words, the response increases whenever the contact time and dose of ferric oxide increase.

**Fig. 4:**
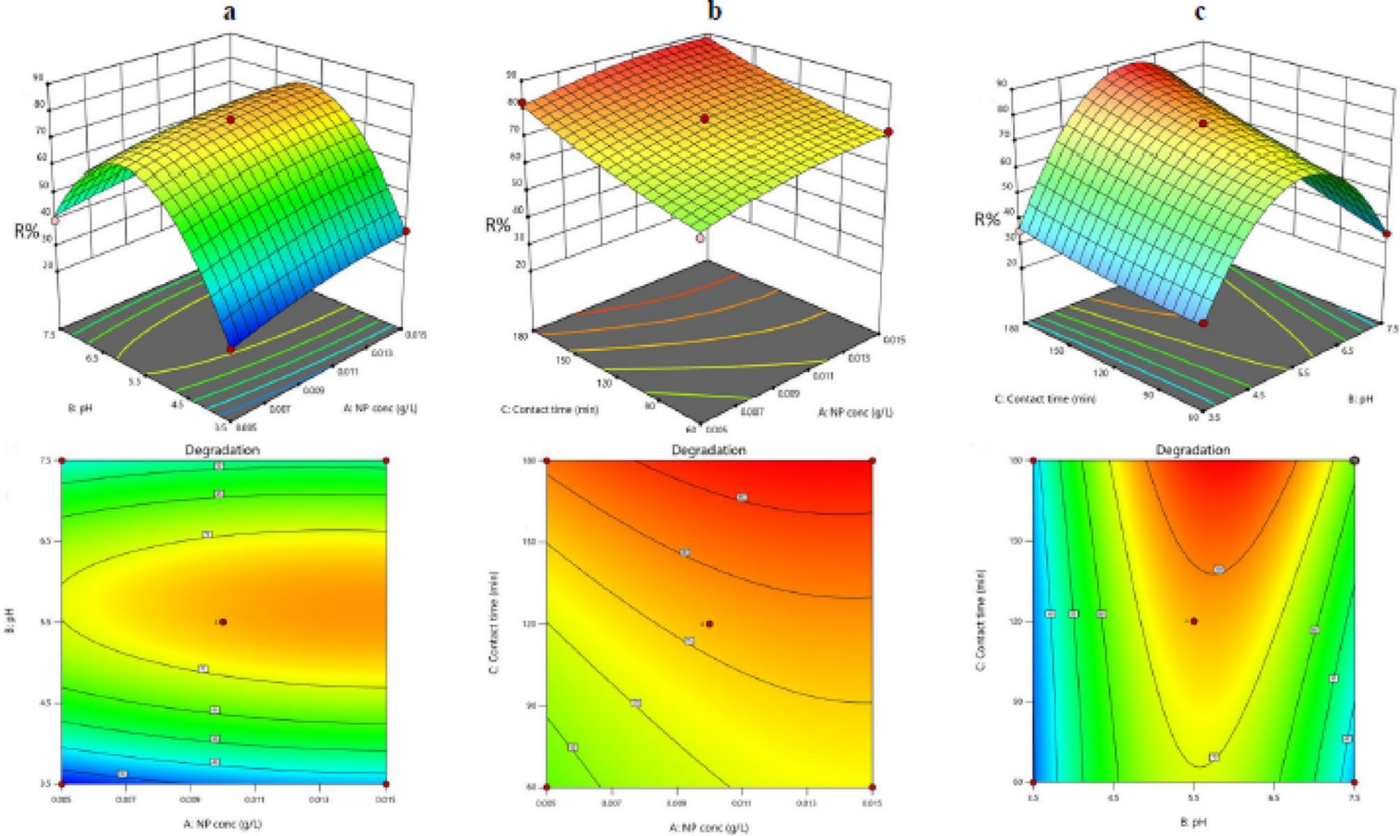
3D and 2D response surface contour plots showing the removal % of CEF: **a** effects of pH and the dose of nanoparticles, **b** effects of the dose of nanoparticles and contact time, **c** effects of contact time and pH

The I-optimal design showed the optimum response was not reached by the simple increase of these factors, but there is a point at which the response reaches the maximum by choosing the best level of each factor as revealed in the 3D structures and 2D contour plots.in Fig. 4, which illustrates the correlation among the predictable variables and response while one of the factors is constant. The curvature in the contour plots signifies the non-linear influences of the variables on the adsorption response for CEF.

Figure 4a shows the interaction between the pH and the dose of ferric oxide, where the removal % was found to be maximized at a higher dose of ferric oxide nanoparticles at 0.015 g/L and pH 5.5 at a constant contact time of 120 min. Figure 4b shows the interaction between the dose of ferric oxide and contact time, where the removal % was found to be exaggerated at a higher dose of ferric oxide and a higher contact time at fixed, where the removal % was found to give good results at moderate pH (5.9) and higher contact time (180 min) at a constant dose of ferric oxide which was 0.013 g/L.

### Adsorption kinetics and isotherm models

**Fig. 5:**
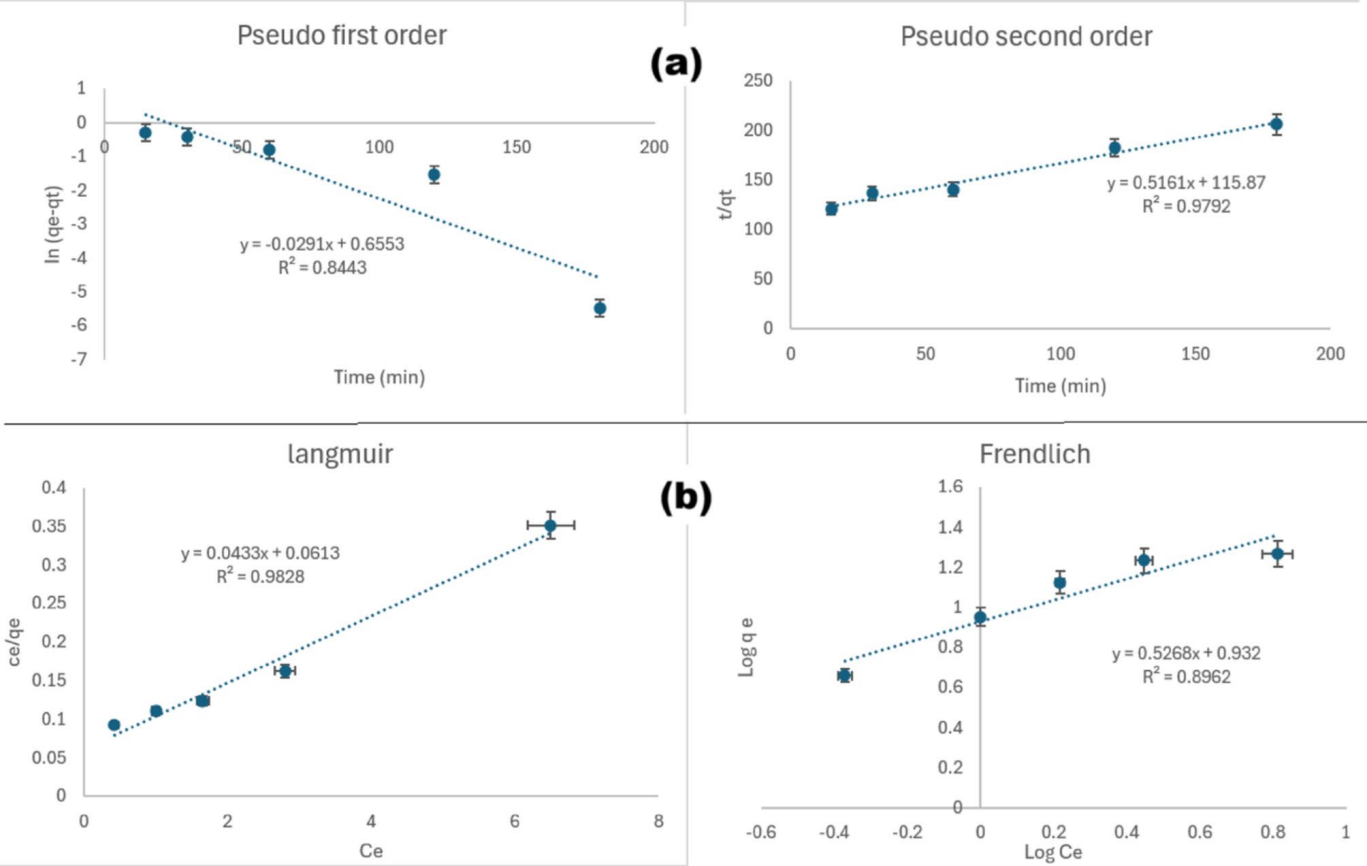
**a** Pseudo first-order and pseudo second-order kinetic models for adsorption of CEF on Fe_3_O_4_ NPs, **b**) Langmuir nd Freundlich adsorption isotherms for adsorption of CEF on Fe_3_O_4_ NPs

The adsorption rate was anticipated using two kinetic models. The adsorption kinetics were investigated using pseudo first-order and pseudo second-order models and are shown in Fig. 5a. The kinetic parameters and equations are listed in Table 4. The pseudo second-order kinetics model was found to be a better match for the presented kinetic data, with an R^2^ of 0.9792, which is higher than the R^2^ calculated for the pseudo first-order kinetics (0.8443), suggesting that chemisorption was the rate limiting step for the adsorption process.

**Table 4:** Equations and parameters of kinetics and isotherm models of CEF adsorption on Fe_3_O_4_

Model	Parameter	Value
Kinetics		
Pseudo first-order ln(qe - qt) = lnqe − k_1_ t	qe (mg/g)	1.926
	k_1_ (/min)	0.0291
	R^2^	0.8443
Pseudo second-order t/qt = t/qe + 1/k_2_ qe^2^	qe (mg/g)	1.938
	k_2_ (/min)	0.0023
	R^2^	0.9792
Isotherms		
Langmuir Ce/qe = Ce/qm + 1/K_L_ qm	q_max_ (mg/g)	23.095
	k_L_ (L/mg)	0.706
	R^2^	0.9828
Freundlich lnqe = lnK_F_ + *n* − 1 lnCe	n	1.898
	k_F_ [(L/g)(mg/g)]^1/n^	8.550
	R^2^	0.8962

It is necessary to explain the adsorption isotherm to show how adsorbate interacts with the adsorbent. The adsorption process was assessed using the Langmuir and Freundlich isotherm adsorption models as shown in Fig. 5b, and the estimated isotherm parameters from the two plots were summarized in Table 4. The collected data showed that Langmuir’s hypothesis best describes the adsorption behavior of CEF molecules on the adsorbent across the tested concentration range due to its higher determination coefficient value (R^2^ = 0.9828). On the other hand, a lower R^2^ value of (0.8962) was calculated for the Freundlich isotherm model. Langmuir’s results demonstrate that active sites are distributed uniformly across the adsorbent where a monolayer of CEF molecules adhere to the NPs.

The previous findings regarding kinetics and isotherm models agrees with reported studies that dealt with the adsorption of a similar cephalosporin (cefoperazone) using Fe_3_O_4_ NPs [52]. All kinetics and isotherm models were investigated at room temperature to minimize energy consumption and maintain sustainability of the treatment and analysis processes.

### Greenness assessment of the HPLC method

Green chemistry principles have been raised due to the growth of ecosystem concerns. It became essential to evaluate the chemists’ actions that can affect the environment commercially and experimentally [53]. Galuszka redefined the twelve principles of green chemistry to fit the analytical chemistry techniques targeting procedures that are eco-friendly with high reliability and accurateness for analysis of biological fluids and pharmaceuticals [54]. The chromatographic technique should be evaluated by greenness assessments because of the use of high-energy instruments and organic solvents [55–57]. The greenness assessments were done using three tools; Analytical Eco-scale, Analytical Greenness (AGREE), and Green Analytical Procedure Index (GAPI).

Analytical Eco-scale [57] (AES) was modified by Galuszka to assess the analytical method, it was based on allocating penalty points, based on hazards of reagents, energy, waste production, and occupational hazard. Penalty points are calculated for the analytical method parameters and then deducted from 100 to compute the eco scale which ranges from 0 to 100. The ideal eco-scale green method will score 100 points, excellent > 70, acceptable > 50, and inadequate will score less than 50 [58]. Our method scored 82 which means that this method is green as shown in Table 5.

**Table 5:**
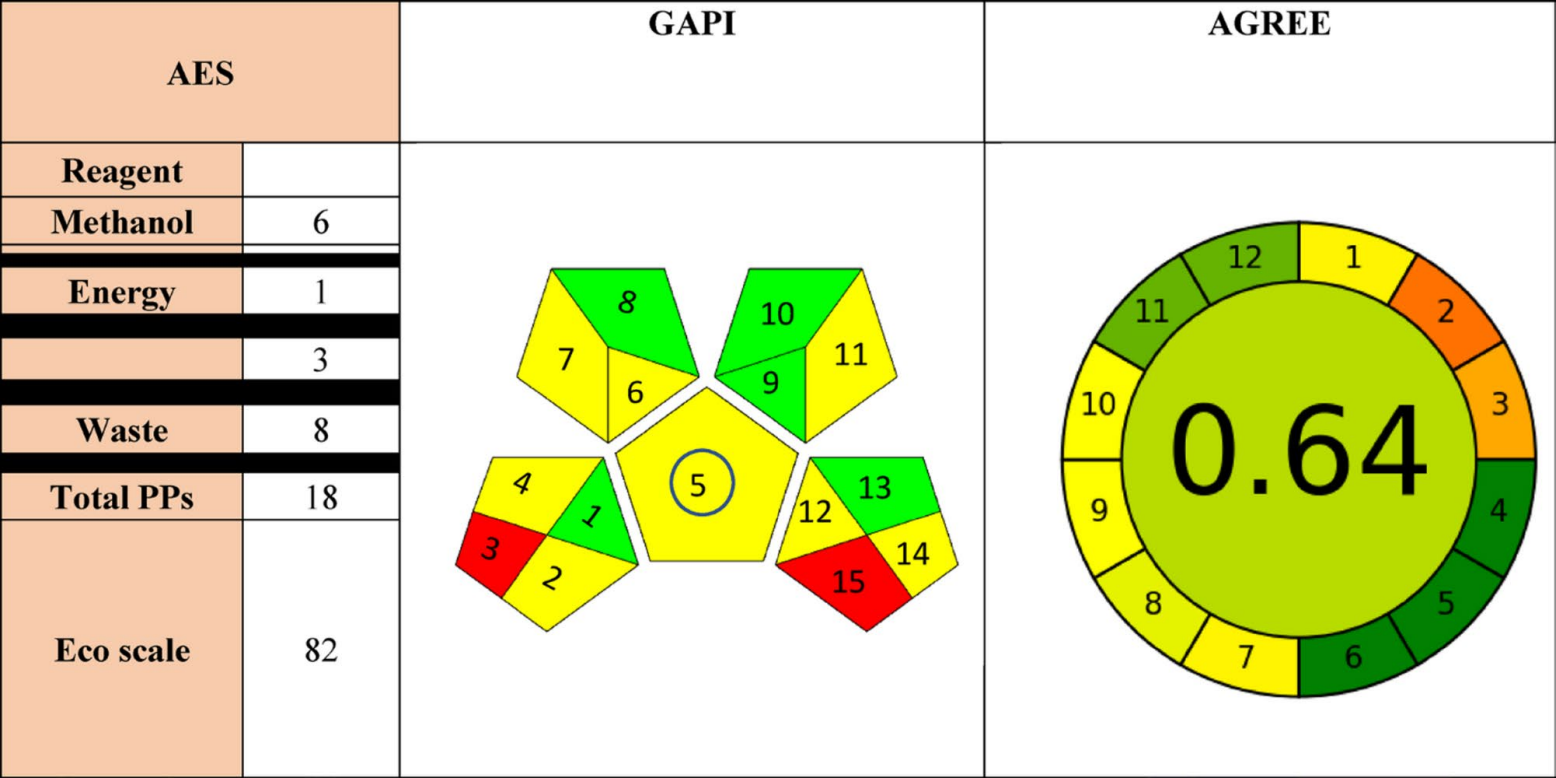
Greenness assessment of HPLC method

GAPI was first introduced by Waslyka as a greenness tool represented by 5 pentagrams that assess 15 analytical aspects including reagents, instrumentation, environmental hazard, waste amount, chemical health, and energy [59]. The process is appraised using three shades green, yellow, and red which are used to assess the effect of each parameter from low, medium, and high accordingly [58]. As shown in Table 5, the pictogram denoted red by the numbers 3 and 15 shows that transportation is required and there is no waste treatment accordingly, while the green color shown in pictograms numbers 1, 8, 9, 10, and13 reflects that the sample was collected in line, no additional treatment was required, amount of solvent used was less than 10 mL, slightly toxic and minimum occupational hazard. Finally, the yellow color reflected in numbers 2, 4, 5, 6, 7, 11, 12 and 14 represents that the preservation was done physically, stored under normal conditions, the method is simple, and micro-extraction and green solvents were used. The detailed GAPI report is listed in Table S2.

AGREE (Analytical greenness) is a comprehensive, flexible greenness tool that develops a colored pictogram which is subdivided into 12 sections, its score ranges from 0 to 1 in which 1 is considered the highest score. The effect of each criterion was marked by color ranging from deep red (lowest) and deep green (highest) based on its positive impact on the environment [58]. Our proposed method scored 0.64 as shown in Table 5, and shifted towards the green color which shows the greenness of the developed method with a high score shaded by green color in 4, 5, 6, 11 and 12 pentagrams regarding the sample preparation stages, automation, and miniaturization, derivatization, toxicity, and operator safety; followed by low scores shaded by yellow color in 1, 7, 8, 9, 10 pentagrams regarding sample treatment, waste, analysis throughput, energy consumption and source of reagents. The lowest scores are shaded by red color in 2 and 3 pentagrams reflecting the sample amount and device positioning. The detailed AGREE report is listed in Table S2.

KPI standards highlight the importance of decision-making when choosing a suitable analytical technique [60]. The strengths and weakness points were considered for the proposed method against reported HPLC [61] and spectrophotometric methods [62]. HPLC methods transcend in terms of selectivity, robustness, accuracy, decision, and applicability KPI [1–4]. The proposed HPLC method exceeded the reported one in terms of sensitivity as it showed lower LOD and LOQ values. Regarding KPI from 5 to 7, the proposed method included a comprehensive greenness assessment which was absent in both reported methods so the sustainability score of the proposed method was found to be 5 which exceeded both reported methods as shown in Table S3.

### Analysis of spiked wastewater

Distilled water was spiked with the amount of 20 ppm of cefixime. Using Derringer’s desirability algorithm, the optimal conditions of removal were suggested to be the dose of Fe_2_O_3_ (13 mg/L), pH (5.9), and a contact time of (180 min) as shown in Figure S5, with a predicted removal percentage of 89.47% with a desirability value of (1.00). By applying the experimental optimum conditions to simulated wastewater, the observed removal percentages were found to be 87.55% % with a bias of 1.9%. Thus, the predicted value was found to be of low prediction error. A comparison was conducted between the proposed method and the other reported ones [63–65] in terms of sensitivity and removal accuracy. The results showed that the proposed method showed a higher removal and lower bias percentage, as shown in Table S4.

## Conclusion

This study described the removal of cefixime from wastewater by adsorption with the aid of ferric oxide nanoparticles. The effective parameters were selected by screening factorial design, which was found to be the dose of ferric oxide nanoparticles, contact time, and pH. The I-optimal design was done to get the optimal conditions for cefixime removal from wastewater, which were recommended to be pH (5.9), the dose of Fe_2_O_3_ (0.013 g/L), and a contact time of 180 min. By applying those conditions, the observed removal percentages were found to be 86.55%. The study also described the development of a chromatographic method for cefixime for monitoring its removal using Kinetex C_18_ as the stationary phase, methanol, and phosphate buffer as the mobile phase (adjusted to pH 6.8) in the ratio of 25:75 v/v, the injection volume was 20 µL and the quantification was done by using the peak area with UV detection at 288 nm. The established method proved to be precise, accurate, rapid, specific, and simple by applying the validation parameters according to the ICH guidelines. The HPLC determination of the adsorbed samples was performed with good resolution and adequate retention time. Moreover, this study was subjected to greenness assessment by using several concepts such as AES, GAPI, and AGREE, showing that this method is green and eco-friendly. The method can be easily applied for large-scale water treatment because it doesn’t require serious pH adjustments or temperature control, and the particles can be separated by applying a magnetic field.

## Supplementary Information

Supplementary Material 1

## Data Availability

The datasets used and/or analyzed during the current study are available from the corresponding author upon reasonable request.
