# Supplementary material for: Response surface optimized removal of cefixime from wastewater samples using magnetic ferric oxide nanoparticles
Source: BMC Chem. 2025 Oct 6;19(1):274. doi: 10.1186/s13065-025-01635-7 (PMC12502269; doi:10.1186/s13065-025-01635-7)
Supplement: Supplementary file 1 — Supplementary Material 1 [file 13065_2025_1635_MOESM1_ESM.docx]

**Supplementary Materials for**

“Response Surface Optimized Removal of Cefixime from Wastewater Samples Using Magnetic Ferric Oxide Nanoparticles”

Rana W. Gaber^1^, Amr M. Mahmoud^2*^, Sarah S. Saleh^1*^

^1^ Analytical Chemistry Department, Faculty of Pharmacy, October University for Modern Sciences and Arts (MSA) 11787 6th October City, Egypt

^2^ Pharmaceutical Analytical Chemistry Department, Faculty of Pharmacy, Cairo University, Kasr-El Aini Street, Cairo, 11562, Egypt

* Corresponding authors’ emails: (A.M.M: [amr.bekhet@pharma.cu.edu.eg](mailto:amr.bekhet@pharma.cu.edu.eg); S.S.S: [sssaleh@msa.edu.eg](mailto:sssaleh@msa.edu.eg))


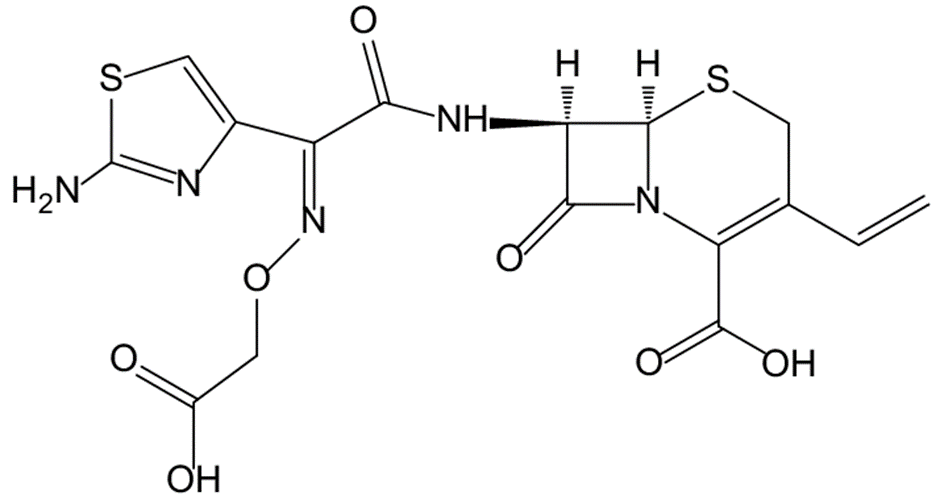


**Fig.S1. Chemical structure of CEF**

**Fig S2. CEF Calibration curve in the concentration range of 10-50 µg/ mL plotted against peak area.**


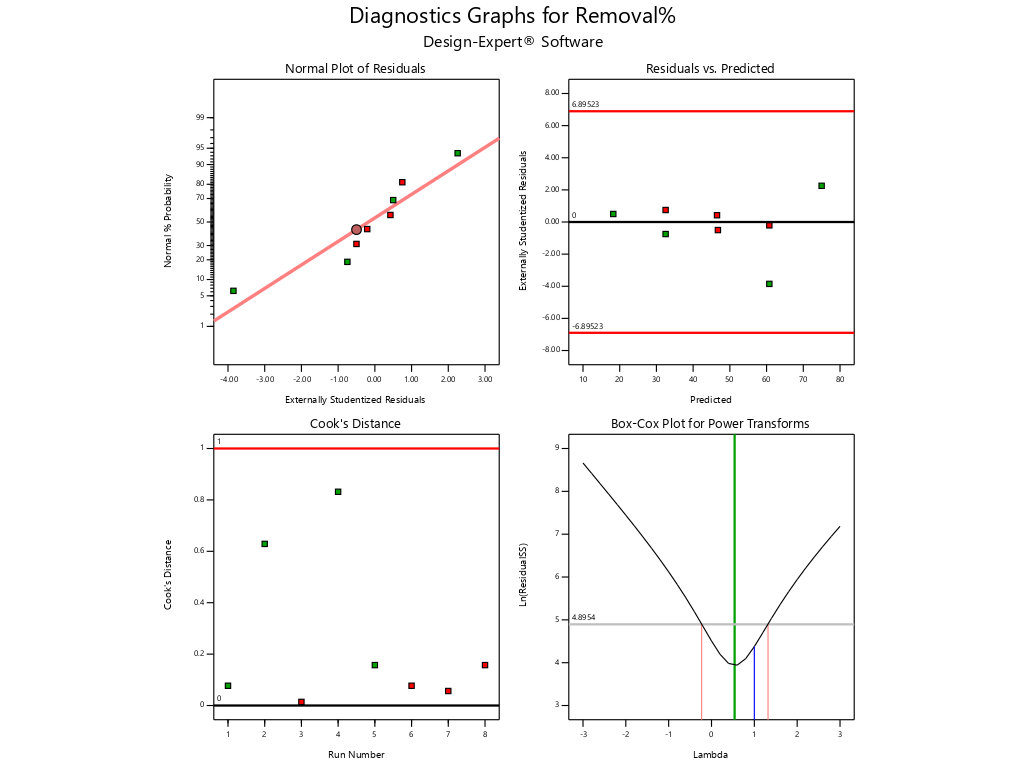


D

C

B

A

**Fig S3. Diagnostic graphs of screening fractional factorial design (A) shows the Normal plot of residuals (Externally Studentized Residuals vs Normal % Probability), (B) shows the Residuals vs. predicted (Predicted vs Externally Studentized Residuals), (C) shows the cook’s distance (Run Number vs Cook's Distance) & (D) shows the box-cox plot for power transformers (Lambda vs Ln(ResidualSS))**


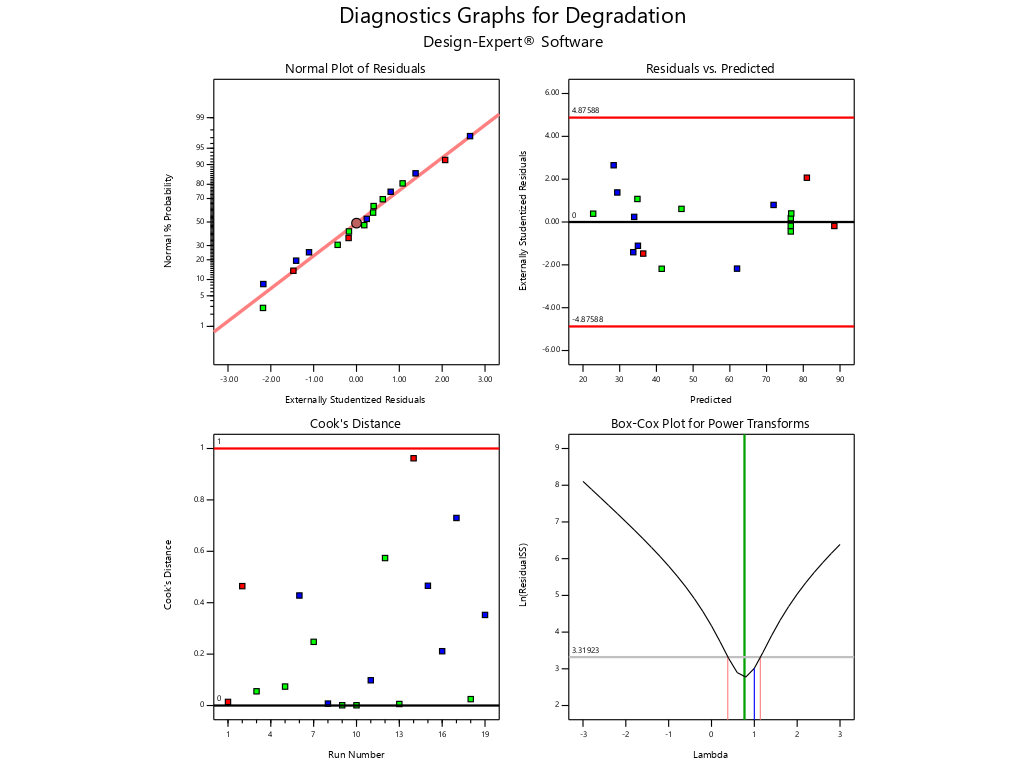


D

C

B

A

**Fig S4. Diagnostic graphs for i-Optimal design for Degradation** **(A) shows the Normal plot of residuals (Externally Studentized Residuals vs Normal % Probability), (B) shows the Residuals vs. predicted (Predicted vs Externally Studentized Residuals), (C) shows the cook’s distance (Run Number vs Cook's Distance) & (D) shows the box-cox plot for power transformers (Lambda vs Ln(ResidualSS))**


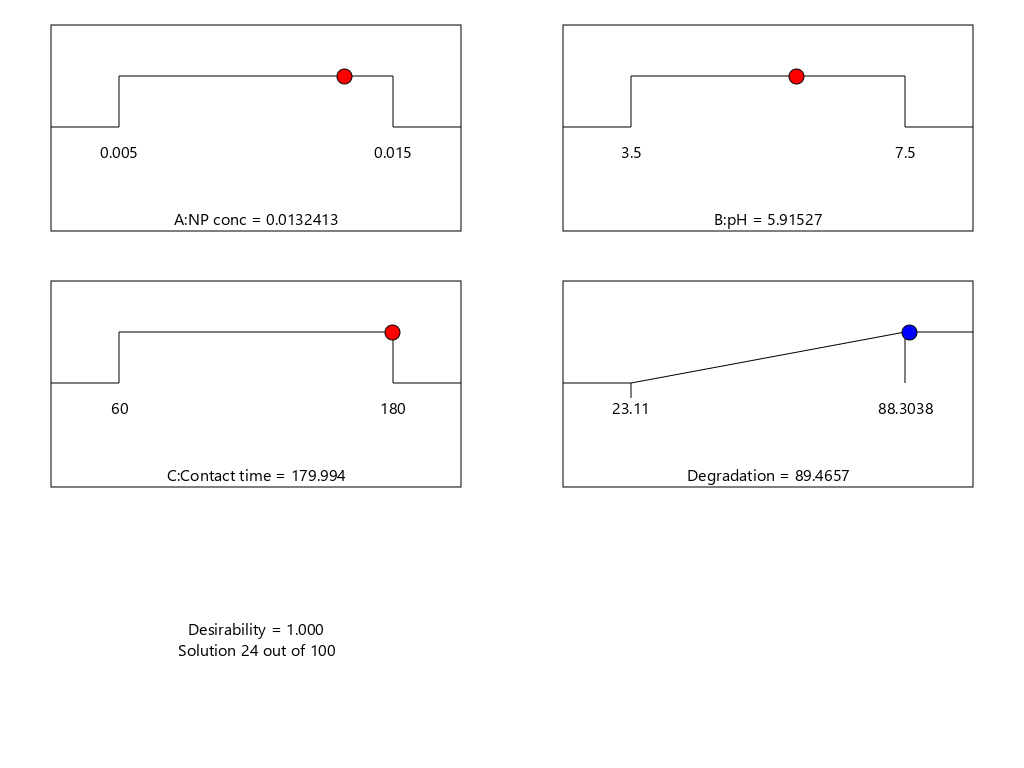


**Figure S5. Solution ramps for optimum removal conditions of CEF with a desirability index of 1.00**

**Table S1. Nanoparticles synthesis**

| Points of comparison | Fe_3_O_2_ | TiO_2_ | rGO |
| --- | --- | --- | --- |
| Synthesis | It was prepared by the chemical co-precipitation approach to produce bare magnetic particles. 23.64 gm of ferric chloride hexahydrate powder (FeCl_3_.6H_2_O) and 9.94 gm of ferrous chloride tetrahydrate powder (FeCl_2_.4H_2_O) were dissolved in 300 mL distilled water. The temperature was raised to 80°C, 80 mL of ammonium aquation (25%) was added under high-speed stirring (350 rpm) to reach pH value 10, this reaction took place for 45 min after that the magnetic nanoparticles were separated by centrifugation 15000 rpm for 15 mins and washed by ethanol and deionized water several times until the pH value was 10 | Anatase particles were prepared by precipitation from homogeneous solution using titanium (IV) isopropoxide (Sigma-Aldrich) as precursor in aqueous solution acidified with nitric acid to pH 2 using a water-to-titanium mole ratio of about 200 | NaBH_4_ aqueous solution added to graphene oxide (GO), The solution was magnetic stirring at 300 rpm for 2 hours at 80 ^o^C. Then, the colloidal was washed with DI water and filtered. The left-over material at the membrane was dried at 80^o^C overnight |
| Size and shape | The nanoparticles appeared to be in black powder form and of size less than 10 nm | The nanoparticles appeared to be spherical like shape powder in anatase phase and of size less than 10 nm. | The nanoparticles appeared to be in black powder sheets and of length 1 – 3 µm, thickness: 1 – 5 nm. |

**Table S2. AGREE and GAPI reports.**

| **AGREE Report** | **GAPI Report** |
| --- | --- |
| **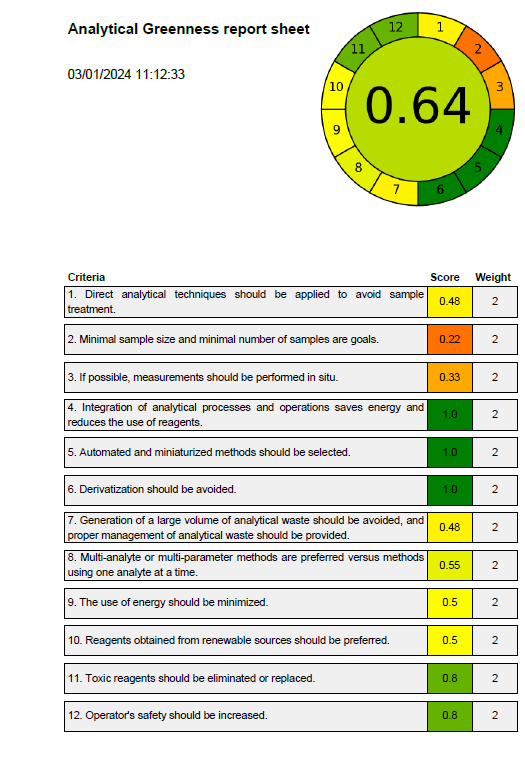** | **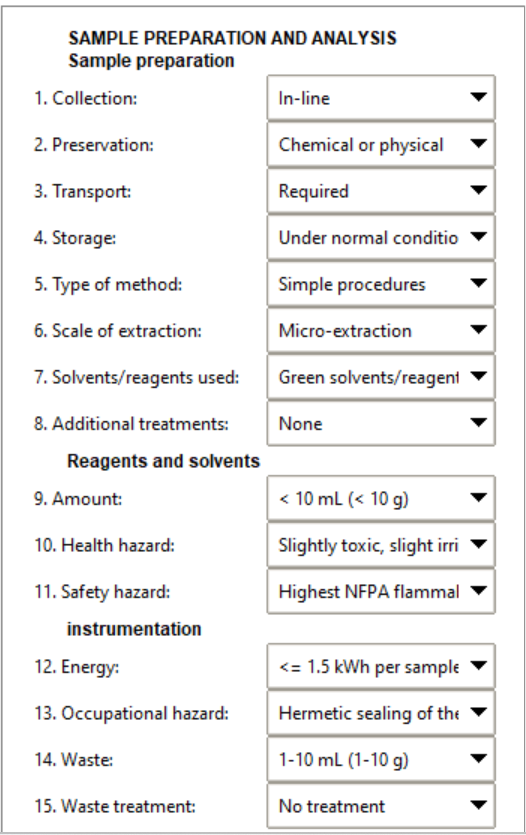** |

**Table S3. Applying KPI standards on proposed and reported methods.**

| **No of standard** | **The standard name** | **Proposed RP-HPLC method** | **Reported RP-HPLC method (60)** | **Reported spectrophotometric method (61)** |
| --- | --- | --- | --- | --- |
| **Validation-bases KPI- standards** | | | | |
| KPI 1 | Selectivity & robustness |  |  |  |
| KPI 2 | Sensitivity |  |  |  |
| KPI 3 | Accuracy and precision |  |  |  |
| KPI 4 | Applicability |  |  |  |
| **Total score for the validation study** | | **4** | **3** | **2** |
| **Sustainability-bases KPI- standards** | | | | |
| KPI 5 | RGB12 Algorithm |  |  |  |
| KPI 6 | Greenness |  |  |  |
| KPI 7 | Blunesses |  |  |  |
| **Total score for sustainability study** | | **1** | **0** | **0** |
| **Final score** | | **5** | **3** | **2** |

**Table S4. Comparison between the removal and bias percentage between the proposed method and other reported methods (62-63).**

|  | **Removal percentage (Sensitivity)** | **Bias Percentage (Accuracy)** |
| --- | --- | --- |
| The proposed method | 86.55% | 1.9% |
| Removal of Cefixime from Water Using Rice Starch by Response Surface Methodology (62) | 70.22% | 6.4% |
| Removal of cefixime cefixime using heterogeneous fenton catalysts: Alginate/magnetite hydroxyapatite nanocomposite (63) | 84% | NA |
| Photocatalytic Degradation of Cefixime Trihydrate by Bismuth Ferrite Nanoparticles (64) | 75% | 1.8% |
